# Environmental Monitoring of PAHs Exposure, Biomarkers and Vital Status in Coke Oven Workers

**DOI:** 10.3390/ijerph17072199

**Published:** 2020-03-25

**Authors:** Luigi Vimercati, Lucia Bisceglia, Domenica Cavone, Antonio Caputi, Luigi De Maria, Maria Celeste Delfino, Vincenzo Corrado, Giovanni Maria Ferri

**Affiliations:** 1 Interdisciplinary Department of Medicine, Occupational Medicine “B. Ramazzini”, University of Bari Medical School, 11 G. Cesare Square, 70124 Bari, Italy; domenica.cavone@uniba.it (D.C.); antonio.caputi@live.it (A.C.); luigi.demaria@uniba.it (L.D.M.); maria.delfino@uniba.it (M.C.D.); vincenzo.corrado@uniba.it (V.C.); giovannimaria.ferri@uniba.it (G.M.F.); 2 Strategic Regional Health and Social Agency of Puglia (AReS Puglia), 52 G. Gentile Street, 70126 Bari, Italy; l.bisceglia@arespuglia.it

**Keywords:** cohort, coke plant, biomarkers, vital status, occupational exposure, sister chromatid exchange, urinary 1-hydroxypyrene (1-OHpy), PAH DNA adducts, total nitro-PAH hemoglobin adducts

## Abstract

A follow-up study of a cohort of workers from a coke plant compared with a control group from the same industrial area was conducted in 2019. The recruitment and environmental and biomarker measurements were performed during 1993/1994. The environmental concentrations of polycyclic aromatic hydrocarbons (PAH), B(a)P, pyrene and nitro-PAH were measured. Personal data were collected via an individual semi-structured questionnaire by a trained physician. All biomarkers were measured after a specific blood drawing for every test. Significant risks (ORs) were observed for nitro-PAH (*≥*0.12 µg/m^3^) [OR = 7.96 (1.01–62.82)], urinary 1-hydroxypyrene (1-OHpy) (≥0.99 µmoles/moles of creatinine) [OR = 11.71 (1.47–92.90)], PAH DNA adducts (P^32^) (≥2.69 adducts/10^8^ nucleotides) [OR = 5.46 (1.17–25.58)], total nitro-PAH hemoglobin adducts (≥161.68 fg/µg of Hb) [OR = 5.92 (1.26–27.86)], sister chromatid exchange (SCE) with TCR (≥377.84 SCE/cell chromosomes) [OR = 13.06 (3.95–93.10)], sister chromatid exchange with T (≥394.72 total SCE) [OR = 13.06 (3.95–93.10)], and sister chromatid exchange with X (≥8.19 mean SCE) [OR = 13.06 (3.95–93.10)]. Significant risk of death for all causes and chromosomal aberrations (48 h) (OR = 7.19 [1.19–43.44]) or micronuclei in culture at 48 h (OR = 3.86 [1.04–14.38]) were also found.

## 1. Introduction

### 1.1. Coke Plants and PAHs

During the production of coke, large quantities of coke oven gas are emitted, resulting in consequent environmental pollution and exposure of both workers and the general population, similar to other productive activities [1,2,3]. The working environment at a coke plant can negatively affect the workers exposed to coke oven emissions (COE) containing polycyclic aromatic hydrocarbons (PAHs), which are formed and released into the environment via the pyrolysis of coke. Both benzo[a]pyrene and pyrene are representative PAHs [4], and the coke oven emissions contribute significantly to the severe pollution of volatile organic compounds (VOCs) in the air [5,6,7,8]. Some studies have shown that PAH exposure from the coke plant still poses a health risk for workers and the general population [1,9]. Coke oven workers at risk should be monitored for adverse effects due to long-term exposure [10]. Coke plant workers are simultaneously exposed to a mixture of aromatic and polycyclic hydrocarbons present in the breathing zone air. Exposure levels are significantly influenced by job categories. Coke byproduct workers are exposed to high levels of benzene, toluene, and xylene and lower levels of PAHs. Coke oven workplaces (top side, coke side, and push side) are characterized by a higher carcinogenic risk than other coke plant workplaces [11]. PAHs in the air of different production sectors in coking plants were measured, and the results showed that the total PAH concentrations ranged from 11.75 to 46.66 micrograms/m^3^; the benzo(a)pirene (BaP) concentration was 0.050–1.054 micrograms/m^3^ in the following areas in descending order: the outlet of the coke oven, the top of the coke oven, the gate, and the point of coke flame out. BaP pollution in the air in the top and outlet of the coke oven was much heavier than that in the soot and air of the arterial road [12]. In coke oven workers, significantly increased death rates for lung cancer and nonmalignant respiratory disease were found. Relatively high air concentrations in mask and urinary 1-HP concentrations have been identified, underlining the need to closely supervise workers wearing respirators [13]. The use of helmet respirators has effectively controlled the long-term average exposure to PAHs for most workers on coke ovens since 1982 [14].

### 1.2. Organ Dysfunctions and Diseases of Coke Oven Workers

Long-term exposure to COE increases the risk of liver dysfunction [15]. A potential risk of sperm dysfunction should be considered for workers occupationally exposed to high levels of PAHs. Cigarette smoking may aggravate this risk [16]. Preventive strategies require the identification of cancer-susceptible individuals resulting from combinations of carcinogen exposure, cancer-predisposing genes, and lack of protective factors [17]. PAH metabolites were related to a reduction in the heart rate variability (HRV) and atherosclerotic cardiovascular disease (ASCVD), and ASCVD was also affected by HRV. Urinary 1-hydroxynaphthalene (1-OHNa), 2-OHNa, and total PAH metabolites (ΣOH-PAH) were dose-responsive and associated with an increased risk of ASCVD. Occupational exposure to PAHs may increase the risk of ASCVD, which is partially mediated by HRV [18]. Workers exposed to a high-concentration of coke oven emissions are more likely to experience hypertension and abnormal electrocardiographic findings than those exposed to low-concentration coke oven emissions. In addition to lung injury, coke oven emissions also have adverse effects on the cardiovascular system. Therefore, more effective measures are needed to protect the health of coke oven workers [19,20]. High levels of 4-OHPh combined with longer working years or being overweight had a joint effect on the risk of diabetes. Elevated 4-OHPh is dose-responsive and was associated with an increased risk of diabetes in coke oven workers. The risk assessment of diabetes related to occupational PAH exposure should take working years and BMI into consideration [21]. The results indicated that urinary copper and zinc levels were positively associated with the risk of diabetes and hyperglycemia in coke oven workers. [22]. The damage to lung function in coke oven workers was associated with exposure to coke oven emissions, the exposure level of coke oven emissions and exposure time, which are the main factors of coke oven workers in lung function damage, and a positive interaction was detected between the two factors [23,24]. Epidemiological studies in coke oven workers have shown an increased risk for cancer and chronic obstructive pulmonary diseases, but these studies are confounded by multiple industrial exposures, most notably to polycyclic aromatic hydrocarbons that are generated during pet coke production [25]. Long-term exposure to COE increases the risk of an interaction between chronic obstructive pulmonary disease (COPD) and cigarette smoking [26]. Some PAHs, such as benzo(a)pyrene and benzo(a)anthracene, are well-established genotoxic agents. Long-term exposure to PAHs may lead to proliferative cell disorders in humans, predominantly in the skin, lung, and bladder [27].

### 1.3. Biomarkers and Coke Oven Workers

White blood cells and lymphocytes are generally used to monitor occupational exposure to PAHs, and coke oven workers, smokers, and slow acetylators sustain more genetic damage in their lymphocytes LYM-DNA from exposure to carcinogenic PAHs than individuals without these factors [28]. The coke oven workers had a significantly higher risk of DNA damage than did the controls, and dose–response relationships were also found between external exposure (exposure category) or internal doses (urinary 1-hydroxypyrene) and DNA damage [29]. Aberrant DNA methylation is one of the best-known epigenetic changes in human cancers and healthy subjects exposed to carcinogens [30,31]. Another report found that urinary PAH metabolites were significantly elevated in coke oven workers. Smoking can significantly modify the effects of urinary 1-hydroxypyrene on high concentrations of urinary 8-OHdG during coexposure to both light or heavy smoking and high 1-hydroxypyrene levels [32]. Urinary 1-hydroxypyrene is a useful biomarker for evaluating total PAH exposure, and coke oven workers with heavy smoking showed more serious DNA oxidative damage than nonsmokers [33,34,35]. Measurement of BPDE-I-DNA adduct levels in coke plant workers is essential in determining cancer risk due to high exposure to PAHs, particularly B[a]P [29]. The BPDE-albumin adduct is a useful biomarker for monitoring long-term exposure to PAHs, and plasma BPDE-albumin adduct levels were significantly correlated with urinary 1-OHP levels in coke oven workers [36]. Occupational exposure to COE may induce both BPDE-Alb adducts and DNA damage in the lymphocytes of coke oven workers, and these two markers are useful for monitoring exposure to COE in the workplace [37]. Genome-wide association studies (GWAS) have identified multiple single-nucleotide polymorphisms (SNPs) associated with lung cancer. Lung cancer risk-associated SNPs and their correlations with PAH exposure were associated with 8-OHdG levels and Micronuclei (MN) frequency [38]. PAHs are established lung carcinogens and may cause mitochondrial toxicity. PAH exposure and PAH-related nDNA genotoxicity are associated with increased mtDNAcn [39]. DNA methylation was quantitated in LINE-1 and O (6)-methyl-guanine-DNA methyltransferase (MGMT). Hypomethylation of LINE-1 and the MGMT promoter could be used as markers for PAH exposure and merit further investigation [40]. Genetic variations in key nucleotide excision repair (NER) genes, especially in the XPA and XPC genes, may modulate DNA damage levels after exposure to PAHs [41]. The modulation of anti-B[a]PDE-DNA adducts in the lymphocyte plus monocyte fraction (LMF) by GSTM1-null and some low-activity NER genotypes may be considered a potential genetic susceptibility factor capable of modulating individual responses to PAH (B[a]P) genotoxic exposure and the consequent risk of cancer in coke oven workers [42]. Coke oven workers had significantly increased levels of urinary 1-hydroxypyrene and micronucleus frequency and decreased DNA repair capacity (DRC) compared with controls. Significant correlations between DRC and micronucleus frequency were found in coke oven workers and all study subjects but not in controls [43]. The micronucleus (MN) frequency associated with biologically effective doses of polycyclic aromatic hydrocarbons (PAH; anti-benzo[a]pyrene diol-epoxide (B[a]PDE)-DNA) within the same subjects’ peripheral blood lymphocytes (PBLs) was evaluated. The increase in MN frequency is mainly related to the specific anti-B[a]PDE-DNA formation within PBLs of the same subject. The results confirmed that the workers had a higher cancer risk than the controls [44].

Elevated serum Glutathione S-transferase (GSTs) activities and increased oxidative DNA damage were found in coke oven workers. Occupational exposure and smoking interact with each other. Serum GST may be used as a biomarker for assessing the exposure to PAHs. Assays of urinary 8-OHdG may be useful for evaluating the risk of lung cancer in coke oven workers [45]. XRCC1 and GSTP1 polymorphisms might influence the susceptibility to DNA damage in occupational PAH-exposed coke oven workers [46]. Significant associations between genetic polymorphisms in the GSTM1, NQO1, and mEH genes and the risk for chromosomal damage were found in occupational PAH-exposed workers, which was related to the mechanism of PAH carcinogenesis [47]. Smoking coke oven workers with genotypes unfavorable for detoxification of aromatic amines (NAT2-ss) and PAH (GSTM1-null) may have an increased risk of developing bladder cancer [48]. The CYP1A1*2 allelic variant has been associated with elevated urinary 1-hydroxypyrene (1-OHP). Individuals with the (linked) CYP1A1*2 or *3 variant alleles have an increased capacity to activate PAHs from tobacco smoke and occupational exposure and, as a result, have an increased risk for PAH-related cancers, especially certain respiratory cancers [49,50]. Some recent studies have examined the role of polymorphisms linked to DNA repair genes in the modulation of genotoxic risk associated with PAH exposure, both for lifestyle (dietary and smoking behaviour) and for occupational reasons, and underlie individual susceptibility to lung cancer [51]). The urinary mutagenic activity of coke workers is associated with PAH occupation-related urinary excretion of 1-pyrenol. This result demonstrates an occupation-related exposure of the bladder epithelium of coke oven workers to mutagenic PAH metabolites and the increased bladder cancer risk in coke oven workers [52].

In this study, we performed an updated analysis of a cohort of coke oven workers to study the associations among several biomarkers of exposure, biological effective dose, effects, and genetic susceptibility with exposure and vital status.

## 2. Materials and Methods

### 2.1. Study Groups

The first group was recruited from the coke plant of an important steel plant of Taranto in southern Italy through a random procedure. A total of 104 male workers were recruited. The control group of 19 male workers was recruited from another industrial plant in the same area with the same selection method. All recruited subjects were asked to provide informed consent and were submitted to (a) fasting in the morning for the blood draw; (b) collection of extemporaneous urine samples; and (c) data collection through a questionnaire. All these procedures were carried out in the plant sick bay during 1993–1994.

### 2.2. Job Title Description

In the cohort there were subjects with various job titles: charging car operator, pushing car operator, coke guide operator, operator of the lids, gas regulator, temperature operator, barrel attendant, quenching car operator, maintenance technician, and combustion technician, all with possible different levels of exposure. Job titles are described in the supplementary materials (Supplementary Material Text S1).

### 2.3. Environmental Monitoring Measurements

Two campaigns of environmental measures were carried out for the single polycyclic aromatic hydrocarbon (PAH) B[a]P and total PAH and nitro-PAH. The first campaign was carried out in May 1992, and the second campaign was carried out in October 1993. In May 1994, a campaign of environmental measures was carried out within the plant from which “unexposed” workers were recruited. This second group represents related workers from the same industrial area. The withdrawal method was based on Gilian personal samplers. These devices were worn for 6 h by a worker representing every different position in the company. However, some determinations were made with air measurements. The area samplers were placed in the work area and allowed the determination of the time-weighted average of substances spread in the working environment; personal samplers enabled the determination of the average individual weighted concentration. Air samples for the determination of PAH and nitro-PAH were collected via a double-body sampler with a fiberglass membrane and a vial with Amberlite XAD-2 resin both in the breathing zone of workers through personal samplers and from certain sites at the plant. A personal air sampler was also used for non-exposed subjects. From the measured environmental data, average or median values for each measurement group made in each single or merged position were calculated. The values of the environmental measures were used to create (a) three groups of exposure to benzo[a]pyrene: (0 = no exposure; 1 = low–moderate exposure (0.001–2.00 µg/mc); 2 = high exposure (>2.00 µg/mc)); (b) three groups of exposure to PAH with four/seven rings: (0 = no exposure; 1=low–moderate exposure (0.001–17.58 µg/mc); 2 = high exposure (>17.58 µg/mc)); (c) three groups of exposure to nitro-PAH: (0 = no exposure; 1 = low–moderate exposure (0.001–0.054 µg/mc)); 2 = high exposure (>0.054 µg/mc)).

#### 2.3.1. Determination of Urinary 1-Pyrenol

The 1-pyrenol levels in urine samples were determined using the method of Jongeneelen et al. [53]. The complete method was described by Clonfero [54].

#### 2.3.2. Determination of Nicotine and Its Metabolites in Urine Samples

Nicotine and its metabolites were determined using the diethyl thiobarbituric acid (DETB) extraction method [55] based on the Koenig reaction. DETB, used as a condensing agent, yields a pink product that can easily be extracted in ethyl acetate. Optical density was measured spectrophotometrically at a wavelength of 532 nm. Metabolite concentrations were calculated according to the calibration curve of cotinine in an aqueous solution.

#### 2.3.3. DNA Adducts

All subjects were asked not to eat fried, grilled, or barbecued meat during the 24 h before sampling.

Chemicals: Tritiated (±)-r-7,t-8-dihydroxy-t-9,10-oxy-7,8,9,10 tetra-hydro-benzo[a]pyrene (3H-anti-B[a]PDE), with a specific activity of 1,941 mCi/mmol, was obtained from the NCI Chemical Carcinogen Standard Repository (Bethesda, MD, USA). The purity and authenticity of the B[a]P metabolite were determined by the NCI Carcinogen Standard Repository. [32P] ATP (>5000 Ci/mmol) was obtained from Amersham (UK). All other reagents were of the purest grade available. Sample Collections: Peripheral venous blood samples were collected from coke oven workers in heparinized vacutainer tubes after at least three consecutive days of PAH exposure and maintained in refrigeration during transport. The PBLs were isolated on Ficoll separating solution (Seromed, Germany), as previously described [56] and frozen at −20 °C for later DNA isolation.

DNA Isolation: DNA from human PBLs was isolated following the original procedure described by Johns [57] with minor modifications [58]. PAH and B[a]P lymphocyte DNA adducts with p32 post-labelling. DNA adducts were detected essentially as described by Reddy [59], with minor modifications [60]. All determinations were carried out in duplicate [61].

#### 2.3.4. Hemoglobin Nitro-DNA Adduct

We used this method to study nitro-PAH exposure. Five representative mononitrate-PAHs were selected for adduct analysis. These compounds, when adsorbed, are metabolized to nitrous and N-hydroxy derivatives that are linked to hemoglobin and plasma proteins. After hydrolysis of the derived family, the corresponding amino-PAH is released and can be quantified. Hemoglobin adducts have been suggested for the biological monitoring of nitro-PAH.

For the hydrolysis, fifty milligrams of hemoglobin were first fixed and then dissolved in eight milliliters of NaOH at pH 12. It was then proceeded to centrifuge the solution and to extract into test tubes the amines in solid phase. From the test tubes the liquid phase was taken. The absorbed components were eluted in 2 × 750 microliters of ethyl acetate and the solution made to mix with MgSO4. So, the amines were derived with pentafluoro propionic acid anhydride (PFPA). The samples were dehydrated by evaporation and placed in solution with 100 microliters of ethyl acetate. Two microliters of this solution were inserted into a Hewlett-Packard 5988 GC-MS and analyzed in the single ion monitoring (SIM) mode. This analytical procedure required derivation to increase volatility. To do this were used pentafluoro propionic acid anhydride (PFPA) and pentafluoro benzaldehyde. Finally, the five derivatives of PFPA were synthesized and used for the preparation of standard solutions. The detection limits for PFPA-2-Aminofluorene is 100 femtograms and for the others of 1 picogram. The recovery of each of the five amino arenas was 78–89%. Covalently, DNA-bound metabolites are mostly analyzed by immunoassays and ^32^P-postlabeling, with sensitivities down to one adduct in 10^7^–10^10^ normal bases. Gas chromatography-mass spectrometry (GC-MS) is generally used for protein adducts, with the sensitivity down to 0.001 pmol/g protein. Target tissues are usually not available to analyze either one of the two types of adducts, but lymphocytes and blood proteins are accepted as reasonable substitutes.

Although reactions with DNA are considered indicative of genotoxic effects, the analysis of protein adducts has advantages that make it attractive for use in exposure control, particularly because methods for adduct measurements in risk assessment have yet to be developed [62].

#### 2.3.5. Urinary Mutagenicity

Urine samples were collected at the end of the work shift (after at least three consecutive days), as described previously [54]. Mutagenic activity was determined using the micro suspension preincubation assay modified from Ames’ basic procedure [63].

#### 2.3.6. GSTM1 and NAT2

Subjects: This study was carried out on some of the coke oven workers examined in previous works [48]. Each subject was asked to fill in a questionnaire providing details of tobacco smoking habit or passive smoking and type of food consumed during the 24 h before urine sampling, with particular attention to fried, grilled, or barbecued meat.

GSTMI and NAT2 genotyping: At least 1 × 10^6^ frozen mononucleated cells collected from blood were used for DNA extraction with saturated NaCl or Chelex 100 (Bio-Rad, Hercules, CA, USA), as described by Walsh et al. [64]. PCR-based GSTMI and NAT2 genotyping was carried out according to the protocols described by Hou et al. [65]

#### 2.3.7. Flow Cytofluorometry (FCM)

From March 1992 to June 1994, FCM and DNA analysis were performed on urine specimens obtained from 123 healthy subjects, of which 90 had worked in coke ovens for at least 5 years; 48 of these 90 persons also smoked at least 20 cigarettes daily. The clinical characteristics of the subjects enrolled in the study are reported in Table 1.

Samples (100–120 mL) of normally voided urine were utilized for the study. After division into two aliquots, one was destined for DNA analysis, and the other was used for cytological evaluation.

DNA Analysis by FCM: For DNA analysis, the samples of fresh urine (at least 50 mL) were fixed immediately in a solution containing methanol and acetic acid (20:1), in accordance with the technique of Deitch [66].

### 2.4. Cytological Analysis

The specimens for cytological analysis (at last 50 mL) were cytocentrifuged and stained according to the method of Papanicolau [67]. The cytological samples were reviewed by the same cytopathologist and classified as having absent, low, intermediate, or high contents of benign, inflammatory or malignant cells. To evaluate cellular preservation to exclude the possibility of necrosis, we analyzed the cases for the presence or absence of cytomorphological features, such as granular chromatin texture, presence of nucleoli, irregularity of the nuclear membrane, hyperchromatic nuclear chromatin, or cytoplasmic degeneration [68]. These features were evaluated in all aneuploid cases and in a small subgroup of diploid samples. A specimen was considered non-evaluable when too few cells were available in the sample [69].

### 2.5. Urinary Mutagenicity Testing

Urine samples were collected in polyethylene containers at the end of the work shift, after at least three consecutive days of exposure to PAH, frozen at −20 °C and kept in the dark until the moment of analysis.

Sample mutagenic activity was then determined using the micro suspension preincubation assay, consisting of a modification to Ames’ basic procedure [63].

### 2.6. Mutation Frequency

Subjects and cell isolation: Peripheral blood samples were obtained from a population of coke oven and non-oven workers. For the same population, urinary 1-pyrenol concentration and urinary mutagenicity [54] and DNA adduct levels [61]) were determined. The features of the plant, the jobs, and the examined population, as well the environmental sampling and the analytical methods, are detailed in the final report of the research supported by the EC Steel and Goal Commission (Contract 7280-01-04). Details on the blood sampling and peripheral lymphocyte isolation are in Celotti [61].

T-cell cloning and mutagenicity assay. Samples of PBLs from the donors (43 coke oven workers and 26 non -oven workers) were tested for their ability to produce 6-thioguanine (6-TG)-resistant clones (HPRT). To determine the mutation frequency at the HPRT locus, we used the T-cell cloning assay according to Albertini et al. [70], with some modifications [71]).

Twenty-four hours after isolation, the cells were centrifuged, counted, and diluted in growth medium (RPMI 1640, 10% fetal calf serum (PCS), 30% T-cell growth factor (TCGF), 5% human serum (HS), 0.5% PHA-M and 5 U/mL interleukin-2 (IL-2)) containing 12 j x M 6-thioguanine (6-TG). TCGF was prepared from a pool of freshly isolated PBLs derived from two or three donors, following the procedure of He et al. [72]).

Multiplex PCR: Individual exons and flanking sequences were simultaneously amplified from the genomic DNA of 161 mutant clones by multiplex PCR (MP-PCR). The lysate of each clone was prepared from 2–3 × 104 cells, following the method of Fuscoe et al. [73]. Primer sequences, buffer and amplification conditions were described by Gibbs [74] and Òsterholm [75]. The sequences and the names of primer pairs 25 (913 bp), 27 (847 bp), and 31 (355 bp) were described in Morris [76].

RT-PCR: The reverse transcription of HPRT mRNA and PCR amplification of the resultant first-strand cDNA (CTGAG-3J_31, 0.1; J.M of reverse primer 2, (72i) GATAATTTTACTGGCGATGT (702), 200) were carried out for 56 T-lymphocyte mutant clones following the method of Yang et al. [77], with minor modifications [74].

### 2.7. CA, MN (48 h–72 h), MN with Cytochalasin B and SCE

Cytogenetic methods: Cultures for cytogenetic tests were initiated within a few hours after blood collection, and the tests were performed by the method in use in the laboratory (whole blood in RPMI 1640 + 10% fetal calf serum, 2 mM L-glutamine, phytohemagglutinin, penicillin, and streptomycin). For CA and MN in conventional cultures, cells were harvested at 48 and 72 h and stained with 10% Giemsa. SCE cells were harvested at 72 h from cultures treated with BrdU 30 µg/mL and stained by the FPG method. For MN in cytokinesis-blocked cultures, cytochalasin B (Sigma) at a final concentration of 3 µg/mL was added at 44 h, and the cells were harvested at 72 h and stained with 10% Giemsa. For each subject at each culture time, 100 metaphases were counted and scored for CA according to ISCN [78]. For statistical analysis, cells with aberrations were grouped in total abnormal metaphases including and excluding gaps, cells with chromatid breaks and exchanges, and cells with chromosome-type aberrations (acentric fragments, dicentric and ring chromosomes, abnormal monocentric chromosomes). MN in conventional cultures at each culture time was scored in 3000 interphase cells (1000 cells per slide). MN in cytokinesis-blocked cultures was scored in 2000 binucleated cells per subject. SCE was evaluated in 50 metaphases per subject. All scoring was carried out blind, with no knowledge of the exposure status [79]. All the cytogenetic methods were described in a previous study [80].

### 2.8. Vital Status

The vital status, the certificates and causes of death of the deceased subjects was updated to 2020, January through the Regional Assisted Care Registry.

### 2.9. Statistical Analysis

The first step was based on the evaluation of the distribution of all the continuous data to ensure the correct parametric or nonparametric statistical approaches were used. The skewness and kurtosis distributions were evaluated, and as a second step, a log transformation and a cut-off at the 66th percentile were applied because the majority of distributions were not Gaussian and had a high number of outliers. A correlation matrix was calculated for the log-transformed data. Univariate and multivariate logistic regression analyses were carried out. All statistical analyses were carried out using STATA 12 software (STATA Corporation, 4905 Lakeway Drive College Station, TX 77845, USA).

### 2.10. Consent to Participate

Informed and written consent was obtained from all participants. All subjects were informed that data from the research protocol would be treated in an anonymous and collective way, with scientific methods and for scientific purposes in accordance with the principles of the Helsinki Declaration. 

### 2.11. Ethical Committee and Ethical Code 

Ethical approval is not necessary because all medical and instrumental examinations were performed according to Italian law concerning the general rules for occupational hygiene (D.P.R. 303/1956).

## 3. Results

### 3.1. Evaluation of All Continuous Variables

All continuous variables related to environmental PAH and nitro-PAH concentration measurements and to the biomarker values generally showed a non-Gaussian distribution. The differences between the means and the medians were very high for all the environmental measurements except nitro-PAH. Additionally, the skewness values were elevated for PAH (Skew = 2.58; Kurt = 8.95), pyrene (Skew = 2.74; Kurt = 10.43), and B[a]P (Skew = 1.97; Kurt = 6.50) and for all the biomarkers of biologic effective dose and effects excluding chromosome aberrations, sister chromatid exchanges, and alterations of urinary DNA (Supplementary Material Table S1).

### 3.2. Main Characteristics of the Study Groups

A comparison of the main characteristics of the two study groups showed relevant differences related to education (coke oven workers had higher percentages of primary school than the control group), night work shifts (night shifts were performed only by coke oven workers), residency (only coke oven workers resided in the “Taranto - Tamburi” neighborhood (very close to the coke plant)), and smoking habits (coke oven smokers had the highest number of pack-year consumption); no difference was observed for age at visit; very important differences were observed for the “actual (2020).date” age (the percentage of coke oven workers over 65 years of age (76.92%) was significantly higher than that observed in the non-coke oven workers (31.58%)), and the highest job length at visit (1993–1994) was significantly higher for coke oven workers than non-coke oven workers (Table 1). The job title distributions showed a high percentage of machine operator for the coke oven workers. The vital status updated to January 2020 and the causes of death are reported in Table 2 and Table 3. Sixteen subjects died, of which eight were from cancer. Particularly, we observed three examples of lung cancer, one of bladder cancer, one of mediastinum cancer, one of tongue cancer and two tumors of the hemolymphatic system. Thirteen subjects were lost to follow up (Outside Residents: OUTRES).

### 3.3. Environmental Exposure Concentrations, Biomarker Measurements, and Polymorphism Frequencies by Study Groups (Comparison of Means or Proportions)

The means of the main continuous variables of exposure (polycyclic aromatic hydrocarbons (PAHs), benzo[a]pyrene (B[a]P), pyrene, nitro-PAH) were significantly higher in coke oven workers than in non-coke oven workers. Only B[a]P was not different.

The means of the log-transformed environmental measurements of polycyclic aromatic hydrocarbon (PAH) concentrations were significantly higher in the coke oven plant (2.77 ± 0.49 µg/m^3^) than in the non-coke oven plant (2.33 ± 0.65 µg/m^3^), and the nitro-PAH concentrations were significantly higher in the coke oven plant (−2.65 ± 0.85 µg/m^3^) than in the non-coke oven plant (−4.82 ± 0.00 µg/m^3^).

The means of benzo[a]pyrene and pyrene did not show important differences. Benzene, nitrosamine, aliphatic amine, and aromatic amine concentrations were only in the coke plant and were all under the limits (Table 4).

*Exposure biomarkers.* The mean urinary hydroxypyrene (1-OH-pyr) level was significantly higher in the coke oven workers (−0.12 ± 1.40 µmoles/moles of creatinine) than in the non-coke oven workers (−2.74 ± 0.71 µmoles/moles of creatinine) (Table 4).

*Biological effective dose biomarkers.* The mean polycyclic aromatic hydrocarbon (PAH) adducts (p32) were significantly higher in the group of coke oven workers (0.80 ± 0.54 adducts/10^8^ nucleotides) than the group of non-coke oven workers (0.40 ± 0.32 adducts/10^8^ nucleotides).

Additionally, the mean total nitro-PAH hemoglobin adducts (TNPHA) showed a significantly higher value in the coke oven workers (4.36 ± 1.56 fg/µg of Hb) than in the non-coke oven workers (1.52 ± 2.90 fg/µg of Hb) (Table 4).

*Biomarkers of effects.* The sister chromatid exchange (SCE) with X was significantly higher in the coke oven workers (2.02 ± 0.30 mean SCE) than in the non-coke oven workers (1.87 ± 1.20 mean SCE). The other biomarkers did not show significant differences (Table 4). For the genetic susceptibility biomarkers, no significant differences in proportions were observed (Table 4).

### 3.4. Correlations between the Exposure Measurements and Different Biomarkers 

**Figure 1:**
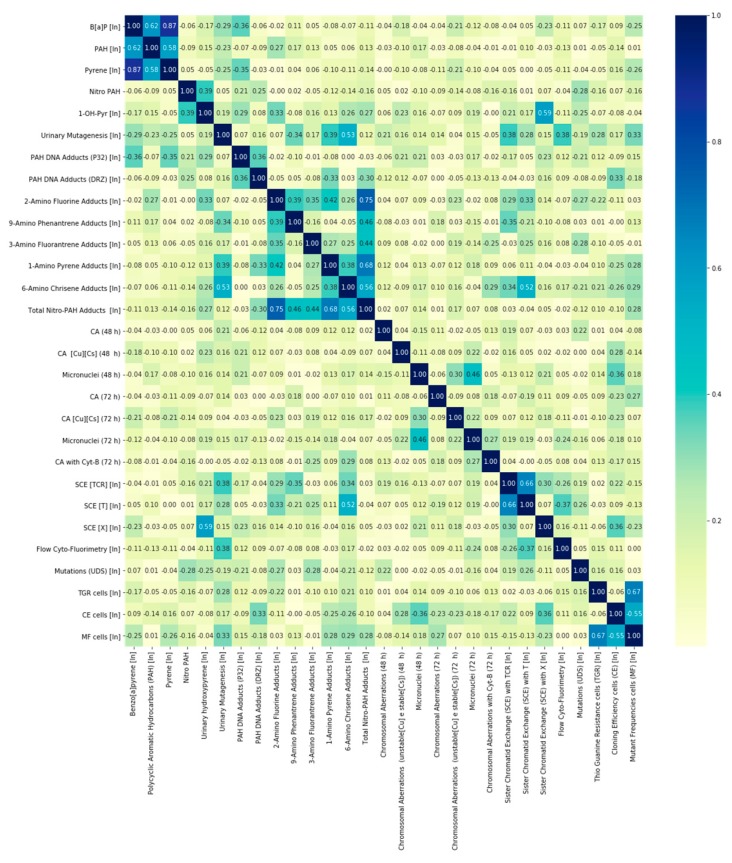
Correlation matrix of ln transformed variables.

Figure 1 shows correlations beetween the exposure measurments and different biomarkers.

#### Exposure Measurements

The correlation matrix shows only a few relationships between exposure measurements and different biomarkers.

Benzo[a]pyrene (B[a]P) environmental measurements were strongly correlated with polycyclic aromatic hydrocarbons (PAHs) [r = 0.62] and pyrene [r = 0.87]. Polycyclic aromatic hydrocarbon (PAH) environmental measurements were strongly correlated with B[a]P [r = 0.62] and pyrene [r = 0.58].

Nitro-polycyclic aromatic hydrocarbon (nitro-PAH) environmental measurements were not correlated with the other environmental measurements.

### 3.5. Biomarkers of Internal Dose

Urinary hydroxypyrene (1-OH-pyr) was strongly correlated with sister chromatid exchange (X) [r = 0.59].

Urinary mutagenesis was strongly associated with 6-amino chrysene hemoglobin adducts (6-ACHA) [r = 0.53]. Relationships of this biomarker with nitro polycyclic aromatic hydrocarbon (nitro-PAH) exposure measurements, biomarkers of biological effective dose and biomarkers of effects were observed.

### 3.6. Biomarkers of Biological Effective Dose

The 2-amino fluorine hemoglobin adducts (2-AFHA) showed a strong correlation with total nitro-PAH hemoglobin adducts (TNPHA) [r = 0.75] and with the other hemoglobin adducts. The 9-amino phenanthrene hemoglobin adducts (9-APhHA) showed a strong correlation only with TNPHA [r = 0.46] and a moderate correlation with 2-AFHA [r = 0.39].

The 3-amino fluoranthene hemoglobin adducts (3-AFlaHA) showed a strong correlation with TNPHA [r = 0.44]. The 1-amino pyrene hemoglobin adducts (1-APHA) showed a strong correlation with TNPHA [r = 0.68] and 2-AFHA [r = 0.42]. The 6-amino chrysene hemoglobin adducts (6-ACHA) showed a strong correlation with TNPHA [r = 0.56], urinary mutagenesis [r = 0.53] and sister chromatid exchange (T) [r = 0.52].

Total nitro-PAH hemoglobin adducts (TNPHA) showed strong correlations with all the hemoglobin adducts. All hemoglobin adducts were correlated to different degrees.

### 3.7. Biomarkers of Effects

As reported in the correlation matrix in Figure 1:

Micronuclei in culture at 48 h (Mic [48 h]) showed a high correlation with the micronuclei in culture at 72 h (Mic [72 h]) [r = 0.46], CE [r = 0.36] and chromosomal aberrations (unstable [Cu] and stable [Cs]) at 72 h (CA Cu-Cs [72 h]) [r = 0.30].

Sister chromatid exchange (SCE) with TCR showed a strong correlation with sister chromatid exchange (SCE) with T [r = 0.66]. Sister chromatid exchange (SCE) with T showed a strong correlation with sister chromatid exchange (SCE) with TCR, as previously reported, [r = 0.66] and with 6-amino chrysene hemoglobin adducts (6-ACHA) [r = 0.52].

Sister chromatid exchange (SCE) with X was highly correlated with urinary hydroxypyrene (1-OH-pyr) [r = 0.59].

Thioguanine resistance (TGR) was strongly correlated with mutation frequencies (MF) [r = 0.67].

Cloning efficiency (CE) was strongly correlated with mutation frequencies (MF) [0.55].

Mutation frequencies (MF) showed a strong correlation with thioguanine resistance (TGR) [r = 0.67]. Effect biomarkers showed slight correlations with exposure measurements, internal dose biomarkers and biological effective dose biomarkers: (1) sister chromatid exchange TGR with 6-amino chrysene hemoglobin adducts; and (2) sister chromatid exchange X with urinary hydroxypyrene (1-OH-pyr), sister chromatid exchange X and polycyclic aromatic hydrocarbons - DNA adducts (DRZ) (PAH-DNA adducts [DRZ]). Many correlations were found among the single effects biomarkers: (1) micronuclei with clonal efficiency and chromosomal aberrations; (2) chromosomal aberrations with 6-amino chrysene hemoglobin adducts and micronuclei; (3) sister chromatid exchange TGR and urinary mutagenesis with 6-amino chrysene hemoglobin adducts; (4) thioguanine resistance (TGR) with mutation frequency; (5) cloning efficiency with mutation frequency, and mutation frequency with thioguanine resistance (TGR) (Figure 1).

Detailed description of the correlation matrix results is given in the supplementary material (Supplementary Material Text S2).

### 3.8. Univariate Risks (ORs) of Observing Values of the Biomarkers Over the 66th Percentiles in the Study Groups (Coke Oven versus Non-Coke Oven Workers)

Coke oven workers showed a high risk (OR = 6.960 [0.99–2.99]) of having measured environmental concentrations of nitro-PAH above the 66th percentile (≥0.12 µg/m^3^), with borderline statistical significance (Supplementary Material Table S2).

Coke oven workers also had significant risks related to the values of biological effective dose biomarkers:

A significant risk of having polycyclic aromatic hydrocarbons (PAH) adducts (p32) ≥ 2.69 adducts/10^8^ nucleotides (OR = 13.22 [1.40–60.01]), a significant risk of observing values of polycyclic aromatic hydrocarbons (PAH) adducts (DRZ) above the 66th percentile (≥1.37 adducts/10^8^ nucleotides) (OR = 3.91 [1.02–22.01]) and a risk of observing values of total nitro-PAH hemoglobin adducts (TNPHA) above the 66th percentile (≥161.68 fg/µg of hg) (OR = 5.31 [1.15–49.33]) (Supplementary Material Table S2).

Coke oven workers also had significant risks related to biomarkers of effects:

A significant risk of observing values of sister chromatid exchange (SCE) with TCR ≥ 377.34 SCE/cell chromosomes (OR = 14.33 [4.08–56.09]), a significant risk of observing values of sister chromatid exchange (SCE) with T ≥ 394.72 total SCE (OR = 14.33 [4.08–56.09]), a significant risk of observing values of sister chromatid exchange (SCE) with X ≥ 8.19 mean SCE (OR = 14.33 [4.08–56.09]) (Supplementary Material Table S2).

Coke oven workers did not show significant risks of observing the presence of polymorphisms of MSPI, ILEVAL, GSTM1, and NAT2 (Supplementary Material Table S2).

### 3.9. Univariate Risks (ORs) to Observe Values of the Biomarkers over the 66th Percentiles in the Study Groups (Dead versus Alive Workers). Risk of Death for all Causes

Among the environmental exposure measurements, only polycyclic aromatic hydrocarbons (PAHs) (≥15.18 µg/m^3^) showed an increased but not significant risk of death (OR = 1.47 [0.42–4.30]).

Among the biomarkers of biological effective dose, only two showed high but not significant risks of death: (1) 2-amino fluorine hemoglobin adducts (≥42.02 fg/µg of Hb) (OR = 2.70 [0.79–10.55]); (2) total nitro-PAH hemoglobin adducts (≥161.68 fg/µg of Hb) (OR = 2.14 [0.63–7.14]) (Supplementary Material Table S3).

The following biomarkers of effects showed high risks with borderline statistical significance: (1) mutation frequencies (MF) (≥25.48 MF/10^8^ cells) (OR = 4.52 [0.95–42.60]); (2) sister chromatid exchange (SCE) with T (≥394.72 total SCE) (OR = 5.84 [0.81–254.08]); and (3) micronuclei in culture at 48 h (Mic [48 h]) (≥2.00 cells (%) with micronuclei) (OR = 3.03 [0.88–11.82]). Instead, there was a significant risk of observing values above the 66th percentile only for chromosomal aberrations (48 h) (CA [48 h]) (≥1 cell total cells with CA) (OR = 5.48 [1.15–51.49]), which is a biomarker of effect (Supplementary Material Table S3).

Among the biomarkers of genetic susceptibility, only GSTM1 (Glutathione S-Transferase Mu 1 gene polymorphisms) (presence) resulted in a high risk of death (OR = 4.61 [0.86–45.95]) (Supplementary Material Table S3).

### 3.10. Univariate Risks (ORs) of Observing Values of the Biomarkers over the 66th Percentiles in the Study Groups (Workers Who Died from Cancer versus Alive Workers)

The environmental exposure measurements showed a moderate but not significant adjusted risk of death for cancer for polycyclic aromatic hydrocarbons (PAH) [≥15.18 µg/m^3^] (OR = 1.87 [0.44–7.98]), and biomarkers of exposure were not associated with an adjusted risk of death for cancer (Supplementary Material Table S4).

Among the biomarkers of biological effective dose, only two showed high but not significant risks of death: (1) 2-amino fluorine hemoglobin adducts (≥42.02 fg/µg of Hb) (OR = 3.50 [0.66–18.55]); (2) 9-amino phenanthrene hemoglobin adducts [≥16.00 fg/µg of Hb] (OR = 2.47 [0.47–12.98]); and (3) total nitro-PAH hemoglobin adducts (≥161.68 fg/µg of Hb) (OR = 2.02 [0.47–8.64]) (Supplementary Material Table S4).

The following biomarkers of effects showed high risks with borderline statistical significance: (1) chromosomal aberrations (48 h) (CA [48 h]) biomarker (≥1 cell total cell with CA) (OR = 5.01 [0.57–43.48]); (2) mutation frequencies (MF) (≥25.48 MF/10^8^ cells) (OR = 4.18 [0.48-36.08]); (3) cells with thioguanine resistance (TGR) (≥2.36 TGR cells/106 cells) (OR = 4.02 [0.46-34.74]); (4) micronuclei in culture at 48 h (Mic [48 h]) (≥2.00 cells (%) with micronuclei) (OR = 3.90 [0.73-20.70]); (5) cells with cloning efficiency (CE) (≥13.30%) (OR = 3.73 [0.43–32.16]) (Supplementary Material Table S4).

Among the biomarkers of genetic susceptibility, only GSTM1 (glutathione S-transferase Mu 1 gene polymorphisms) was present in all those who died from cancer.

### 3.11. Multivariate Risks (ORs) of Observing Values of the Biomarkers over the 66th Percentiles in the Study Groups (Coke Oven versus Non-Coke Oven Workers)

Coke oven workers showed an adjusted significant high risk (OR = 7.96 [1.01–62.82]) of observing measured *environmental concentrations of nitro-PAH* above the 66th percentile (≥0.12 µg/m^3^) (Table 5).

Coke oven workers also had significant risks related to biological effective dose biomarkers:

A significant risk of observing values of polycyclic aromatic hydrocarbons (PAH) adducts (p32) ≥ 2.69 adducts/10^8^ nucleotides (OR = 5.46 [1.17−25.58]), a high risk of observing values of polycyclic aromatic hydrocarbons (PAH) adducts (DRZ) above the 66th percentile (≥1.37 adducts/10^8^ nucleotides) (OR = 3.36 [0.89–12.65]), and a significant risk of observing values of total nitro-PAH hemoglobin adducts (TNPHA) above the 66th percentile (≥161.68 fg/µg of hg) (OR = 5.92 [1.26–27.86]) (Table 5).

Coke oven workers also had significant risks related to biomarkers of effects: a significant risk of observing values of sister chromatid exchange (SCE) with TCR ≥ 377.34 SCE/cells chromosomes (OR = 13.06 [3.95–43.16]), a significant risk of observing values of sister chromatid exchange (SCE) with T ≥ 394.72 total SCE (OR = 13.06 [3.95–43.16]), and a significant risk of observing values of sister chromatid exchange (SCE) with X ≥ 8.19 mean SCE (OR = 13.06 [3.95–43.16]) (Table 5).

Coke oven workers did not show significant risks of observing the presence of polymorphisms of MSPI, ILEVAL, GSTM1, and NAT2. Only for NAT2 was a high risk with borderline statistical significance observed (OR = 3.02 [0.79–11.56]) (Table 5).

### 3.12. Multivariate Risks (ORs) of Observing Values of the Biomarkers over the 66th Percentiles in the Study Groups (Dead versus Alive Workers)

The environmental exposure measurements and biomarkers of biologic effective dose were not associated with adjusted risk of death for all the causes. Among the biomarkers of biological effective dose, only two showed high but not significant risks of death: (1) 2-amino fluorine hemoglobin adducts (≥42.02 fg/µg of Hb) (OR = 2.49 [0.72–8.61]); (2) total nitro-PAH hemoglobin adducts (≥161.68 fg/µg of Hb) (OR = 2.38 [0.69–8.24]) (Table 6). The following biomarkers of effects showed high risks with borderline statistical significance: (1) mutation frequencies (MF) (≥25.48 MF/10^8^ cells) (OR = 3.12 [0.57–16.82]); (2) sister chromatid exchange (SCE) with T (≥394.72 total SCE) (OR = 3.81 [0.44–32.95]); (3) cells with thioguanine resistance (TGR) (≥2.36 TGR cells/106 cells) (OR = 2.41 [0.55–10.57]); (4) cells with cloning efficiency (CE) (≥13.30%) (OR = 2.90 [0.63–19.85]). Instead, there was a significant risk of observing values above the 66th percentile only for chromosomal aberrations (48 h) (CA [48 h]) (≥1 cell in total cells with CA) (OR = 7.19 [1.19–43.44]) and micronuclei in culture at 48 h (Mic [48 h]) (≥2.00 cells (%) with micronuclei) (OR = 3.86 [1.04–14.38]) (Table 6). Among the biomarkers of genetic susceptibility, only GSTM1 (Glutathione S-Transferase Mu 1 gene polymorphisms) (presence) was related to a high risk of death (OR = 3.24 [0.52–20.00]) (Table 6).

### 3.13. Multivariate Risks (ORs) of Observing Values of the Biomarkers over the 66th Percentiles in the Study Groups (Workers Who Died from Cancer versus Alive Workers)

The environmental exposure measurements showed a moderate but not significant adjusted risk of death for cancer for nitro-PAH (≥0.12 µg/m^3^) (OR = 1.61 [0.23–10.93]), and biomarkers of exposure were not associated with an adjusted risk of death for cancer (Table 7).

Among the biomarkers of biological effective dose, only three showed high but not significant risks of death: (1) 2-amino fluorine hemoglobin adducts (≥42.02 fg/µg of Hb) (OR = 3.60 [0.62–20.86]); (2) 9-amino phenanthrene hemoglobin adducts [≥16.00 fg/µg of Hb] (OR = 2.70 [0.0.46–15.66]); and (3) total nitro-PAH hemoglobin adducts (≥161.68 fg/µg of Hb) (OR = 1.70 [0.35–8.04]) (Table 7).

The following biomarkers of effects showed high risks with borderline statistical significance: (1) chromosomal aberrations (48 h) (CA [48 h]) (≥1 cell of total cells with CA) (OR = 6.61 [0.66–65.41]); (2) micronuclei in culture at 48 h (Mic [48 h]) (≥2.00 cells (%) with micronuclei) (OR = 3.67 [0.65–20.62]) (Table 7).

Among the biomarkers of genetic susceptibility, only GSTM1 (glutathione S-transferase Mu 1 gene polymorphisms) was present in all those who died from cancer.

The positive predictive values (PPVs) and the specificity of most of the biomarkers studied were not significant for all causes of death (Supplementary Material Tables S5 and S6).

## 4. Discussion

We observed significant risks (ORs) between the coke oven workers and nitro-PAH [OR = 7.96 (1.01–62.82)], urinary 1-hydroxypyrene (1-OHpy) [OR = 11.71 (1.47–92.90)], PAH DNA adducts (P^32^) [OR = 5.46 (1.17–25.58)], total nitro-PAH hemoglobin adducts [OR = 5.92 (1.26–27.86)], sister chromatid exchange (SCE) with TCR [OR = 13.06 (3.95–93.10)], sister chromatid exchange with T [OR = 13.06 (3.95–93.10)], and sister chromatid exchange with X [OR = 13.06 (3.95–93.10)].

Strong but not significant associations were found between the coke oven workers and indicators of exposure to pyrene, cells with diagonal radioactive zone adducts (DRZ), and the presence of polymorphisms of NAT2. The positive predictive values (PPVs) and the specificity of most of the biomarkers studied were very high only for the coke oven workers.

Significant risk of death for all causes and chromosomal aberrations (48 h) (OR = 7.19 [1.19–43.44]) and micronuclei in culture at 48 h (OR = 3.86 [1.04–14.38]) were also found. We also observed significant associations between the coke oven workers and the levels of different biomarkers (nitro-IPA, urinary hydroxypyrene, DNA adducts, hemoglobin adducts, exchanges between sister chromatids (SCE), chromosomal aberrations (AC)) and deaths due to all causes.

Strong but not significant associations were found between the workers of the coking plants, the measures of exposure to pyrene, the cells with diagonal radioactive zone adducts (DRZ), the presence of NAT2 polymorphism, the cells with thioguanine resistance (TGR), the cells with cloning efficiency (EC), and all-cause mortality.

### 4.1. Relationship between Different Biomarkers and Exposure to PAH and PAH Derivatives of Coke Workers

The results of our study are in agreement with several studies relating to the exposure of coke oven workers to PAHs, and other professional and non-professional factors have been reported in the literature and their associations with exposure, internal dose, biological effective dose, effect, and genetic susceptibility biomarkers. Wang et al. in 1998 [81] investigated the toxicological genetic effects on workers exposed to polycyclic aromatic hydrocarbons (PAHs) and sister chromatid exchanges (SCEs). The results showed that the SCE in the coke oven workers was significantly higher than that of the controls. Chen et al. in 2003 [82] evaluated the relationship between the levels of DNA-PAH adducts in workers with occupational exposure to coke oven emissions divided into three exposure groups based on the job: top side workers, workers on the machine, and staff in the factory offices. The authors found a significant correlation between the urinary 1-OHP levels of the exposed groups and their levels of DNA-PAH adducts. The logistic regression model revealed that the levels of DNA-PAH adducts were significantly different from one job category to another. Pavanello et al. in a 2005 study [42] evaluated the influence of four polymorphisms of nucleotide excision repair genes (NER) (xeroderma pigmentosum-C (XPC) -PAT +/−, xeroderma pigmentosum-A (XPA) 5’; noncoding region-A23G, XPD-exon 23 A35931C Lys751Gln, xeroderma pigmentosum D (XPD) -exon 10 G23591A Asp312Asn) and that of glutathione S-transferase mu1 (GSTM1-active or null) in Polish coking plant workers. The results showed that workers with low DNA repair ability of the XPC-PAT+/ +/+ and XPA-A23A genotypes had significantly increased levels of anti-B[a] PDE-DNA adducts. DNA adducts were also measured in workers without GSTM1 activity (GSTM1-null genotype), workers with unfavorable XPC-PAT+/+/+ and XPA-A23A NER genotypes, alone or in combination with the GSTM1-null genotype had higher levels of adducts. The increase in the levels of anti-B[a] PDE-DNA adducts was significantly correlated in a multiple linear regression analysis to an exposure to PAHs, the lack of activity of GSTM1, and the low DNA repair capacity of the XPC-PAT+/+/+ genotype. Modulation of anti-B[a]PDE-DNA adducts by GSTM1-null and some low activity NER genotypes can be considered to be a potential genetic susceptibility factor capable of modulating individual responses to genotoxic exposure to PAHs (B[a]P). In 2007, Wang et al. [37] recruited 207 male workers exposed to coke oven gas (COE) and 102 controls not exposed to COE in the same steel plant in northern China. The researchers measured concentrations of benzo [a] pyrene-r-7, t-8, t-9, c-10-tetrahydotetrol-albumin (BPDE-Alb) adducts in plasma and DNA damage in peripheral blood lymphocytes. Multivariate logistic regression analysis revealed that the odds ratio (OR) for BPDE-Alb adducts associated with exposure was 1.72 (95% CI 1.06 to 2.81). The results suggest that occupational exposure to COEs can induce both BPDE-Alb adducts and DNA damage in the lymphocytes of coke oven workers and that these two markers are useful for monitoring exposure to COEs in the workplace. In a 2012 study, Bieniek and Łusiak [11] evaluated the exposure of coke oven workers and byproduct workers in Poland to aromatic hydrocarbons (AHs) and polycyclic aromatic hydrocarbons (PAHs). A toxic equivalence factor BaP (eq) was used to estimate the human health risk associated with respiratory exposure to PAHs. The results of personal air measurements (median values of the sum of nine carcinogenic PAHs) were 2.115 μg/m^3^ (coke oven workers, *n* = 207), 0.326 μg/m^3^ (coke byproduct workers, *n* = 33) and 0.653 μg/m^3^ (total area workers, *n* = 38). The equivalent concentrations of benzo[a]pyrene (BaP (eq)) of 10 PAHs were 1.33, 0.183, and 0.284 μg/m^3^, respectively. It has been found that exposure levels are significantly influenced by job categories. Workers of coke byproducts showed significantly more exposure to benzene, toluene, and xylene and less exposure to PAHs than other workers. Coking plant workers are mainly exposed to PAHs. Talaska et al. in 2014 [83] measured the levels of some biomarkers in people with different job duties in a modern coking plant. The average levels of 1-hydroxypyrene (1HP) increased the closer a group of workers was to the kilns and were higher in the upper kilns with an average level of 11.6 μg/L. The levels of 1HP were increasingly found in the following categories of workers: supervisors, zone workers, side kiln workers, upper and side kiln workers, and upper kiln workers. The study conducted by Samir et al. in 2019 [84] aimed to investigate the relationship between the PAH biomarkers 1-hydroxypyrene (1-OHP), DNA adducts, and 8-hydroxy-2-deoxyguanosine (8-OHdG) in coke oven workers. Eighty-five exposed workers and 85 unexposed controls were included in this study. Urinary 1-OHP, 8-OHdG and BPDE-DNA adducts were measured. The average levels of urinary 1-OHP (6.3 μmol/mol creatinine), urinary 8-OHdG (7.9 ng/mg creatinine), and DNA adducts (6.7 ng/μg DNA) in the exposed group were significantly higher than those of the unexposed group. In exposed workers, significant positive correlations were found between the level of 1-OHP and duration of work, 8-OHdG, and the levels of DNA adducts. Workers with higher exposure to PAHs were more prone to oxidative DNA damage.

### 4.2. Relationship between Different Biomarkers and Vital Status

No results were found in the literature related to the validation of these biomarkers towards causes of death, although multiple publications agree with the results of our study. In 1983, Hurley et al. [85] assessed the mortality of coke oven workers in Great Britain by means of two studies with a total of 6767 male workers. The percentage of lung cancer deaths was approximately 20% higher than that of manual workers in general. The excess occurred mainly among younger men. The ratio of lung cancers to all other cancers was also higher than expected. Overall, the lung cancer mortality rates in kiln workers were similar to those of men who do not work in kilns. Additionally, Redmond et al. (1983) found an excessive relative risk for lung cancer in topside coke oven workers with 15 years of exposure or more. There was also evidence for a consistent dose–response relationship in lung cancer mortality when the duration and location of employment at the coke ovens were considered. Bertrand et al. [86] assessed the influence of occupational exposure on respiratory cancer mortality, particularly lung and upper respiratory and digestive tract cancers, in a cohort of 534 male workers from the two coal mine coking plants in Houillères du Bassin de Lorraine who retired from work between 1963 and 1982. Lung cancer mortality was 2.5 times higher than expected. This excess mortality differs between the two coke ovens (standardized mortality ratios of 3.05 and 1.75, respectively). It was not significantly higher in those exposed for more than five years or exposed directly to the ovens or workers near the ovens compared to those exposed for less than five years or to those not exposed at all. Swaen et al. [87] in 1991 studied the mortality of coke workers in the Netherlands. The researchers recruited a group of 11,399 former workers and followed the mortality rates until 1984. Of these workers, 5639 had worked in the coking plant for at least six months between 1945 and 1969. The other 5740 had worked in another factory during the same period and formed an unexposed control group. Significantly higher mortality rates from lung cancer and nonmalignant respiratory diseases were found in the coking workers compared to the control workers. Among tar distillery workers, the lung cancer rate was higher than expected. The risk of gastric cancer and nonmalignant respiratory diseases in workers in the coke shipment department was increased. Similarly, to evaluate the association between a carcinogenic exposure indicator (IPA adducts to peripheral blood leukocyte DNA) and an early indicator of neoplastic transformation (antigens of the epithelial cell membrane of sputum cells), Assennato et al. [88] recruited 350 coke oven workers and 100 unexposed workers. The results showed that smokers, people with reduced lung function, and those with morphological dysplasia of sputum cells had high levels of DNA adducts; however, no significant difference was found between the groups of coke oven workers and unexposed controls. Chaun et al. in a 1993 study [89], focused on the mortality of retired coke oven workers. This study confirmed the increase in lung cancer and showed excess mortality from all causes, including all types of cancer and cardiovascular disease. A significant increase in all-cause deaths, especially those due to lung cancer, colon cancer, and cardiovascular disease, was found for people who worked near ovens. Excess mortality was found for all causes, especially for stomach cancer, even in subjects who worked on the byproducts. Due to the small numbers of subjects, these results were not fully validated. In the same year, Kriek et al. [90] published a study based on the analysis of PAH-DNA adducts to white blood cell DNA. The researchers analyzed the DNA of white blood cells of coke oven workers and workers of an aluminum production plant and showed the presence of IPA-DNA adducts. Forty-seven percent of coke oven workers had detectable levels of IPA-DNA adducts in their white blood cells compared to 27% of the controls. In both groups, smokers had significantly higher levels of IPA-DNA adducts than nonsmokers; however, the total levels of IPA-DNA adducts in the white blood cells of lung cancer patients were much higher than those generally found in healthy smokers. In a 1993 study, Franco et al. [91] recruited a cohort of 538 male workers employed in a coking plant in Carrara in the period 1960-1985. The follow-up period ranged from 1 January 1960 to 31 December 1990. The author observed significant excess lung cancer mortality: 19 deaths were observed against 10.02 deaths predicted using national rates. The results suggest the possible influence of this occupation on lung cancer mortality. Some relevance to mortality from bronchopulmonary tumors was reported in a study conducted by Moulin et al. in 1995 [92] in two factories that produce stainless steel to evaluate the risk of lung cancer among workers employed in coke ovens, blast furnaces, foundries, and electric ovens. The professional exposures of interest were chromium compounds, nickel compounds, polycyclic aromatic hydrocarbons (PAHs), silica, and asbestos. All male workers who had at least one year of employment between 1 January 1960 and 31 December 1990 were followed up for mortality. The cohorts included 6324 (factory 1) and 5270 workers (factory 2). Nonsignificant increases in lung cancer were observed in the coke oven and blast furnace workers, where exposures of interest may have occurred. Occupational exposure to coke oven emissions is associated with significant excess mortality from cancer of the respiratory system and of the prostate. The respiratory cancer risk for coke oven workers is higher than for non-oven workers. Relative risk values for cancer of the prostate ranged as high as 1.93.

In 2007, Birdsey et al. [93] focused on the possible correlation between race, occupation, and lung cancer, confirming these relationships. In fact, black men were at greater risk of lung cancer mortality than white men among 4668 bakers but not among 33,605 employees. The results confirmed a previously demonstrated association between exposure to carcinogenic emissions from coke ovens, race, and mortality from lung cancer. In 2013, Miller et al. [27] assessed the association between lung cancer mortality and exposure to polycyclic aromatic hydrocarbons in British coking plants. Two cohorts, employees of the National Smokeless Fuels (NSF) and the British Steel Corporation (BSC), for a total of over 6600 British coke oven workers employed in 1967, were followed until mid-1987 for mortality. Previous analyses suggested an excess lung cancer risk of approximately 25%; thus, this study was designed to re-analyze existing data on lung cancer mortality, incorporating revised and improved estimates of exposure, especially to benzo[a]pyrene. The analyses have shown mixed results. In all BSC plants, the relative risk coefficient for the work of five or more years on the tops of the ovens, where the exposures were high, was 1.81, a statistically significant result. However, the results for those with less than five years always on the tops of the ovens did not suggest a trend towards increased risk. These results were consistent with an effect of work in the coking plants on the risk of lung cancer, especially work on the tops of the ovens.

Genome-wide association studies (GWAS) have identified multiple single-nucleotide polymorphisms (SNPs) associated with lung cancer. Lung cancer risk-associated SNPs and their correlations with PAH exposure were associated with 8-OHdG levels and MN frequency. In 2014, Dai et al. identified multiple single nucleotide polymorphisms (SNPs) associated with lung cancer in 1557 coke oven workers in China. These researchers established associations between SNPs and genetic damage caused by polycyclic aromatic hydrocarbons (PAHs). Lung cancer risk-associated SNPs and their correlations with PAH exposure were associated with urinary 8-hydroxydeoxyguanosine (8-OHdG) levels and micronucleus (MN) frequency [38].

Li et al. [94] in 2015 published a study that aimed to analyze the potential associations of BMI with levels of chromosomal damage and lung cancer risk. First, 1333 male workers from a coking plant were recruited to examine their levels of chromosomal damage, and then, a cohort study of 12,052 men was performed to investigate the association of BMI with the incidence of lung cancer. In this way, it was found that male workers with excess body weight (BMI ≥ 25 kg/m^2^) had lower MN frequency levels than men with normal weight (BMI between 18.5 and 24.9). This cohort study indicated that the relative risk (RR) for men with BMI ≥ 25 of developing lung cancer was 35% lower than the RR for men of normal weight. Further meta-analyses showed that compared to men of normal weight, men with BMI ≥ 25 had a decreased risk of lung cancer in both East Asian populations and other populations. These results indicate that men with excess body weight have significantly decreased levels of chromosomal damage and a lower risk of lung cancer than those with normal weight.

Singh et al. in 2018 in a meta-analysis found a significant risk of lung cancer RR 1.55 (95% CI 1.01–2.37) among coke workers in spite of the limitations of the data analysis include lack of adjusting for the confounding effects of smoking, and lack of data on exposure levels of polycyclic aromatic hydrocarbons [95].

Yuhang et al. in 2019 revealed the linear dose-effect associations between PAHs exposure and mosaic loss of chromosome Y (mLOY) among 1005 male coke-oven workers [96]. In a 2019 study, Samir et al. [90] recruited 85 exposed Egyptian coke oven workers and 85 unexposed controls to measure urinary 1-OHP, 8-OHdG, and BPDE-DNA adduct levels. The gene expression of CYP2E1 and XRCC1 was also assessed by PCR, with the aim of investigating the relationship between PAH biomarkers and the role of polymorphisms of the cytochrome CYP2E1 gene and of the DNA repair gene XRCC1 in the detection of workers at risk. The levels of 1-OHP, DNA adducts, and 8-OHdG in the exposed group were significantly higher than those of the unexposed group. The carriers of the XRCC1 variant had the highest levels of 1-OHP, DNA adducts and 8-OHdG and the lowest CYP2E1 gene expression. Workers with high exposure to PAHs were more prone to oxidative DNA damage and tumor development.

Our study has some limitations. The small size of the studied cohort, which reduced the statistical power of the analytical evaluations, especially concerning the association between the different types of biomarkers and different job titles or the different types of biomarkers and the vital status of coke oven workers. Thus, we could not determine the association between single causes of death and the biomarkers. Another limitation is the impossibility of evaluating the improvements in the methods of biomarker assessment in the last decades. Due to the limitations of the experimental part related to the old methods of biomarkers assessment, we need to plan further research with a larger cohort and updated methods for the biomarkers determination.

## 5. Conclusions

Despite the limitations of this study, we observed significant associations between coke oven workers and nitro-PAH, urinary hydroxypyrene, DNA-PAH adducts, total nitro-PAH hemoglobin adducts and sister chromatid exchanges (SCEs). We also observed significant associations between coke oven workers, the levels of different biomarkers (nitro-IPA, urinary hydroxypyrene, DNA adducts, hemoglobin adducts, exchanges between sister chromatids (SCE), chromosomal aberrations (AC)), and deaths due to all causes. Additionally, the positive predictive values (PPVs) and the specificity of most of the biomarkers studied were very high for exposure of coke oven workers but not for vital status.

## Figures and Tables

**Table 1:** Frequency distribution of the main individual characteristics by study groups.

Variables		Total Workers		Coke Plant Workers		Non-Coke Plant Workers	
		*n*	%	*n*	%	*n*	%
Education	Elementary school	58	47.15	57	54.81	1	5.26
	Secondary school	48	39.02	36	34.62	12	63.16
	High school	15	12.20	10	9.62	5	26.32
	University	2	1.63	1	0.96	1	5.26
Work shift	Day	29	23.58	10	9.62	19	100
	Day, Afternoon	21	17.07	21	20.19	0	0
	Day, afternoon, Night	73	59.35	73	70.19	0	0
Residency	Taranto Borgo	12	9.76	10	9.62	2	10.53
	Taranto Tamburi-State	12	9.76	12	11.54	0	0
	Taranto San Vito	33	26.83	24	23.08	9	47.37
	Taranto Province	66	53.66	58	55.77	8	42.11
Pack-years	0	28	22.76	20	19.23	8	42.11
	0.1–17.10	53	43.09	44	42.31	9	47.37
	>17.10	42	34.15	40	38.46	2	10.53
Broiled foods	No	8	6.50	8	7.69	0	0
	Yes	115	93.50	96	92.31	19	100
Age at visit	≤35 years	9	7.32	5	4.81	4	21.05
	36–45 years	57	46.34	50	48.08	7	36.84
	>45 years	49	39.84	49	47.12	8	42.11
Age 2019	≤55 years	2	1.63	0	0	2	10.53
	56–65 years	35	28.46	24	23.08	11	57.89
	>65 years	86	69.92	80	76.92	6	31.5
Job length at visit	≤10 years	21	17.07	18	17.31	3	15.79
	11–20 years	67	54.47	53	50.96	14	73.68
	>20 years	35	28.46	33	31.73	2	10.53
Total workers		123	100	104	100	19	100

**Table 2:** Vital status of the study groups at the end of study (2020).

	Coke Oven Workers	Non-Coke Oven Workers	Total
Alive	75	19	94
* Deaths for all the causes	16	0	16
Lost at follow up (OUTRES)	13	0	13
Total	104	19	123

* non cancer diseases (n.6), cancer diseases (n. 8), unknown diseases (n.2).

**Table 3:** Characteristics of Study Death Subject.

ID	Names	Year of Death	Age at Death	Latency	Cause of Death
1	A.C.	2013	62	20	Cancer
2	M.C.	2018	70	25	Unknown
3	S.P.	2009	61	16	Cancer
4	S.V.	2016	66	23	Cancer
5	C.A.	2015	62	22	Non Cancer
6	C.L.	2019	74	26	Cancer
7	B.G.	2017	66	24	Non Cancer
8	P.G.V.	2013	67	20	Non Cancer
9	R.G.	2000	56	7	Cancer
10	C.A.	2008	64	15	Cancer
11	M.G.	2014	67	21	Cancer
12	G.M.	2012	66	19	Cancer
13	I.F.	2008	50	15	Non Cancer
14	C.C.	2002	50	9	Unknown
15	M.E.	2002	55	9	Non Cancer
16	V.M.	2012	68	19	Non Cancer

**Table 4:** Environmental exposure concentrations, biomarkers measurements, and polymorphisms frequencies by study groups.

		Coke Plant Workers			Non-Coke Plant Workers				
VARIABLES (LN)		*n*	Means	s.d.	*n*	Means	s.d.	t	*p*
Environmental exposure	Benzo[a]pyrene (B[a]p)	104	0.034	0.780	19	0.230	0.630	−1.020	0.150
	Polycyclic Aromatic Hydrocarbons (PAH)	104	2.770	0.490	19	2.330	0.650	3.420	0
	Pyrene	104	0.029	0.910	19	−0.008	0.520	0.170	0.420
	Nitro PAH	104	−2.650	0.850	19	−4.820	0	10.980	0
	Benzene	34	3.320	0.540					
	Nitrosamines	34	−4.560	0.210					
	Aliphatic Amines	91	0.830	1.110					
	Aromatic Amines	34	2.300	0					
Biomarkers of exposure	Urinary Hydroxypyrene (p_ 1_OH)	83	−0.120	1.040	18	−2.740	0.710	10.110	0
	Urinary Cotinine	54	−2.060	1.820					
Biomarkers of Biologic Effective Dose	PAH DNA Adducts (P32)	99	0.800	0.540	17	0.400	0.320	2.890	0.002
	Cells with Diagonal Radioactive Zone (DRZ)	85	0.079	0.980	2	0.320	0.360	−0.340	0.360
	2-Amino Fluorene Hemoglobin Adducts	85	2.870	1.680	11	1.200	1.110	3.110	0.001
	9-Amino Phenanthrene Hemoglobin Adducts	73	2.370	1.240	10	1.930	1.190	1.040	0.150
	3-Amino Fluoranthene Hemoglobin Adducts	57	2.340	1.190	6	1.780	1.220	1.090	0.130
	1-Amino Pyrene Hemoglobin Adducts	97	3.090	1.750	17	2.400	1.460	1.540	0.060
	6-Amino Chrysene Hemoglobin Adducts	54	2.250	1.430	4	1.140	0.960	1.510	0.068
	Total Nitro-PAH Hemoglobin Adducts	102	4.360	1.560	19	3.220	1.520	2.900	0.002
Biomarkers of effects	Cells with Chromosomal Aberrations in culture at 48 h	49	0.270	0.380	13	0.210	0.330	0.560	0.280
	Cells with Chromosomal Aberrations (unstable[Cu] e stable[Cs]) at 48 h	38	0.160	0.330	5	0.130	0.300	0.170	0.420
	Cells (‰) with Micronuclei in culture at 48 h	91	0.390	0.570	18	0.210	0.710	1.190	0.110
	Cells with Chromosomal Aberrations in culture at 72 h	45	0.240	0.360	12	0.230	0.340	0.150	0.430
	Cells with Chromosomal Aberrations (Instable(Cu) e stable(Cs)) a 72 ore	40	0.170	0.370	8	0.250	0.350	−0.560	0.280
	Cells (‰) with Micronuclei in culture at 72 h	92	0.590	0.500	19	0.500	0.390	0.700	0.240
	Cells with Chromosomal Aberrations in culture at 72 h with Cytocala B	92	2.590	0.970	19	3.040	0.470	−1.970	0.025
	Sister Chromatid Exchange (SCE) with TCR	32	5.420	0.990	15	5.500	0.780	−0.260	0.390
	Sister Chromatid Exchange (SCE) with T	32	5.570	0.910	15	5.350	0.920	0.770	0.220
	Sister Chromatid Exchange (SCE) with X	32	2.020	0.300	15	1.870	0.120	1.790	0.039
	Alteration of Urinary DNA (Flow Cytofluorometry)	67	1.640	0.350	7	1.720	0.430	−0.540	0.290
	Mutations with Unscheduled DNA Synthesis (UDS)	88	6.510	0.780	6	8.430	0.260	−5.940	0
	Cells with Thioguanine Resistance (TGR)	46	0.480	0.880	18	0.570	1.070	−0.340	0.360
	Cells with Cloning Efficiency (CE)	47	2.200	0.840	18	2.290	0.540	−0.400	0.340
	Cells with Mutant Frequencies (MF)	48	2.770	1.040	18	2.770	0.990	−0.010	0.490
**VARIABLES (%)**		***n*. tot.**	***n*. polymorphisms**	**%**	***n*. tot.**	***n*. polymorphisms**	**%**	**X^2^**	***p***
Biomarkers of genetical susceptibility	MSPI (CYPA1A variant Restriction enzyme polymorphism)	76	16	76.190	18	5	23.810	0.370	0.530
	ILEVAL (CYPA1A variant Isoleucine-Valine polymorphisms)	76	14	82.350	18	3	17.650	0.030	0.860
	GSTM1 (Glutathione S-Transferase Mu 1 gene polymorphisms)	76	42	79.250	18	11	20.750	0.200	0.650
	NAT2 (N-acetyltransferase 2 gene polymorphisms)	73	49	90.740	11	5	9.260	1.950	0.162

**Table 5:** Distribution of adjusted estimates of risks (*ORs) to observe values of different biomarkers higher to the 66th percentile among coke oven plant workers versus non-coke oven plant workers.

Variables		ORs	LCI (95%)	UCI (95%)
Environmental exposure	Benzo[a]pyrene (B[a]p) [≥1.40 µg/m^3^]	0.61	0.22	1.71
	Polycyclic Aromatic Hydrocarbons (PAH) [≥15.18 µg/m^3^]	1.21	0.42	3.49
	Pyrene [≥1.60 µg/m^3^]	1.90	0.61	5.93
	Nitro PAH [≥0.12 µg/m^3^]	7.96	1.01	62.82
Biomarkers of exposure	Urinary Hydroxypyrene (p_ 1_OH) [≥0.99 µmoles/moles of creatinine]	11.71	1.47	92.90
	Urinary Cotinine [≥0.38 µmoles/moles of creatinine]	0.06	0.00	0.54
Biomarkers of Biologic Effective Dose	PAH DNA Adducts (P32) [≥2.69 Adducts/10^8^ nucleotides]	5.46	1.17	25.58
	Cells with Diagonal Radioactive Zone (DRZ) [≥1.37 Adducts/108 nucleotides]	3.36	0.89	12.65
	2-Amino Fluorene Hemoglobin Adducts [≥42.02 fg/µg of Hb]	1.18	0.42	3.30
	9-Amino Phenanthrene Hemoglobin Adducts [≥16.00 fg/µg of Hb]	0.78	0.27	2.23
	3-Amino Fluoranthene Hemoglobin Adducts [≥17.24 fg/µg of Hb]	0.48	0.15	1.52
	1-Amino Pyrene Hemoglobin Adducts [≥41.70 fg/µg of Hb]	1.40	0.47	4.13
	6-Amino Chrysene Hemoglobin Adducts [≥16.00 fg/µg of Hb]	0.41	0.12	1.38
	Total Nitro-PAH Hemoglobin Adducts [≥161.68 fg/µg of Hb]	5.92	1.26	27.86
Biomarkers of effects	Cells with Chromosomal Aberrations in culture at 48 h [≥1 Total cells with CA]	0.54	0.18	1.60
	Cells with Chromosomal Aberrations (unstable[Cu] e stable[Cs]) at 48 h [≥1 Total cells with CA]	2.22	0.72	6.86
	Cells (‰) with Micronuclei in culture at 48 h [≥2.00 cells (‰) with micronuclei]	1.34	0.47	3.82
	Cells with Chromosomal Aberrations in culture at 72 h [≥1 Total cells with CA]	0.57	0.20	1.62
	Cells with Chromosomal Aberrations ((Instable(Cu) e stable [Cs]) a 72 ore [≥1 Total cells with CA]	1.20	0.43	3.36
	Cells (‰) with Micronuclei in culture at 72 h [≥2.33 cells (‰) with micronuclei]	2.08	0.67	6.42
	Cells with Chromosomal Aberrations in culture at 72 h with Cytocala B [≥22.60 cells (‰) with micronuclei/total cells]	0.59	0.20	1.76
	Sister Chromatid Exchange (SCE) with TCR [≥377.84 sce/cells chromosomes]	13.06	3.95	43.16
	Sister Chromatid Exchange (SCE) with T [≥394.72 total sce]	13.06	3.95	43.16
	Sister Chromatid Exchange (SCE) with X [≥8.19 mean sce]	13.06	3.95	43.16
	Alteration of Urinary DNA (Flow CytoFluorimetry) [≥5.90]	0.30	0.09	1.02
	Mutations with Unscheduled DNA Synthesis (UDS) [≥1149.72 cpm/10^6^ cells]	0.03	0	0.21
	Cells with Thioguanine Resistance (TG^R^) [≥2.36 TG^R^ cells/10^6^ cells]	2.05	0.73	5.77
	Cells with Cloning Efficiency (CE) [≥13.30%]	1.90	0.67	5.34
	Cells with Mutant Frequencies (MF) [≥25.48 MF/10^6^ cells]	2.42	0.85	6.87
Biomarkers of genetical susceptibility	MSPI (CYPA1A variant Restriction enzyme Polymorphism) [Presence]	0.71	0.21	2.41
	ILEVAL (CYPA1A variant Isoleucine-Valine polymorphisms) [Presence]	1.42	0.34	5.85
	GSTM1 (Glutathione S-Transferase Mu 1 gene polymorphisms) [Presence]	0.78	0.21	2.80
	NAT2 (N-acetyltransferase 2 gene polymorphisms) [Presence]	3.02	0.79	11.56

**Table 6:** Distribution of adjusted estimates of risks (*ORs) to observe values of different biomarkers higher to the 66th centile by vital status (deaths for all the causes).

Variables		ORs	LCI (95%)	UCI (95%)
Environmental exposure	Benzo[a]pyrene (B[a]p) [≥1.40 µg/m^3^]	0.72	0.18	2.88
	Polycyclic Aromatic Hydrocarbons (PAH) [≥15.18 µg/m^3^]	0.82	0.14	4.82
	Pyrene [≥1.60 µg/m^3^]	0.44	0.09	2.04
	Nitro PAH [≥0.12 µg/m^3^]	0.74	0.16	3.45
Biomarkers of exposure	Urinary Hydroxypyrene (p_ 1_OH) [≥0.99 µmoles/moles of creatinine]	1.15	0.35	3.72
	Urinary Cotinine [≥0.38 µmoles/moles of creatinine]	1.56	0.36	6.75
Biomarkers of Biologic Effective Dose	PAH DNA Adducts (P32) [≥2.69 Adducts/10^8^ nucleotides]	0.90	0.27	2.97
	Cells with Diagonal Radioactive Zone (DRZ) [≥1.37 Adducts/108 nucleotides]	0.66	0.18	2.36
	2-Amino Fluorene Hemoglobin Adducts [≥42.02 fg/µg of Hb]	2.49	0.72	8.61
	9-Amino Phenanthrene Hemoglobin Adducts [≥16.00 fg/µg of Hb]	1.59	0.47	5.36
	3-Amino Fluoranthene Hemoglobin Adducts [≥17.24 fg/µg of Hb]	0.53	0.15	1.83
	1-Amino Pyrene Hemoglobin Adducts [≥41.70 fg/µg of Hb]	1.35	0.40	4.48
	6-Amino Chrysene Hemoglobin Adducts [≥16.00 fg/µg of Hb]	0.83	0.22	3.02
	Total Nitro-PAH Hemoglobin Adducts [≥161.68 fg/µg of Hb]	2.38	0.69	8.24
Biomarkers of effects	Cells with Chromosomal Aberrations in culture at 48 h [≥1 Total cells with CA]	7.19	1.19	43.44
	Cells with Chromosomal Aberrations (unstable[Cu] e stable[Cs]) at 48 h [≥1 Total cells with CA]	0.44	0.13	1.50
	Cells (‰) with Micronuclei in culture at 48 h [≥2.00 cells (‰) with micronuclei]	3.86	1.04	14.38
	Cells with Chromosomal Aberrations in culture at 72 h [≥1 Total cells with CA]	0.43	0.12	1.52
	Cells with Chromosomal Aberrations ((Instable(Cu) e stable [Cs]) a 72 ore [≥1 Total cells with CA]	0.93	0.27	3.14
	Cells (‰) with Micronuclei in culture at 72 h [≥2.33 cells (‰) with micronuclei]	0.79	0.24	2.60
	Cells with Chromosomal Aberrations in culture at 72 h with Cytocala B [≥22.60 cells (‰) with micronuclei/total cells]	1.14	0.33	3.85
	Sister Chromatid Exchange (SCE) with TCR [≥377.84 sce/cells chromosomes]	1.54	0.29	8.10
	Sister Chromatid Exchange (SCE) with T [≥394.72 total sce]	3.81	0.44	32.95
	Sister Chromatid Exchange (SCE) with X [≥8.19 mean sce]	1.70	0.32	8.90
	Alteration of Urinary DNA (Flow Cytofluorimetry) [≥5.90]	0.36	0.10	1.27
	Mutations with Unscheduled DNA synthesis (UDS) [≥1149.72 cpm/10^6^ cells]	1.55	0.48	5.05
	Cells with Thioguanine Resistance (TG^R^) [≥2.36 TG^R^ cells/10^6^ cells]	2.41	0.55	10.57
	Cells with Cloning Efficiency (CE) [≥13.30 %]	2.90	0.63	19.85
	Cells with Mutant Frequencies (MF) [≥25.48 MF/10^6^ cells]	3.12	0.57	16.82
Biomarkers of genetical susceptibility	MSPI (CYPA1A variant Restriction enzyme Polymorphism) [Precence]	0.63	0.09	4.33
	ILEVAL (CYPA1A variant Isoleucine-Valine polymorphisms) [Presence]	0.14	0.00	2.47
	GSTM1 (Glutathione S-Transferase Mu 1 gene polymorphisms) [Presence]	3.24	0.52	20.00
	NAT2 (N-acetyltransferase 2 gene polymorphisms) [Presence]	0.56	0.12	2.63

**Table 7:** Distributions of adjusted estimates of risks (*ORs) to observe values of different biomarkers higher to the 66th centile by cancer death.

VARIABLES		ORs	LCI (95%)	UCI (95%)
Environmental exposure	Benzo[a]pyrene (B[a]p) [≥1.40 µg/m^3^]	0.77	0.11	5.05
	Polycyclic Aromatic Hydrocarbons (PAH) [≥15.18 µg/m^3^]	1.11	0.11	11.32
	Pyrene [≥1.60 µg/m^3^]	0.39	0.04	3.75
	Nitro PAH [≥0.12 µg/m^3^]	1.61	0.23	10.93
Biomarkers of exposure	Urinary Hydroxypyrene (p_ 1_OH) [≥0.99 µmoles/moles of creatinine]	0.37	0.06	2.16
	Urinary Cotinine [≥0.38 µmoles/moles of creatinine]	0.63	0.12	3.14
Biomarkers of Biologic Effective Dose	PAH DNA Adducts (P32) [≥2.69 Adducts/10^8^ nucleotides]	0.36	0.06	2.10
	Cells with Diagonal Radioactive Zone (DRZ) [≥1.37 Adducts/108 nucleotides]	0.51	0.09	2.87
	2-Amino Fluorene Hemoglobin Adducts [≥42.02 fg/µg of Hb]	3.60	0.62	20.86
	9-Amino Phenanthrene Hemoglobin Adducts [≥16.00 fg/µg of Hb]	2.70	0.46	15.66
	3-Amino Fluoranthene Hemoglobin Adducts [≥17.24 fg/µg of Hb]	0.89	0.18	4.36
	1-Amino Pyrene Hemoglobin Adducts [≥41.70 fg/µg of Hb]	1.61	0.34	7.49
	6-Amino Chrysene Hemoglobin Adducts [≥16.00 fg/µg of Hb]	0.66	0.13	3.28
	Total Nitro-PAH Hemoglobin Adducts [≥161.68 fg/µg of Hb]	1.70	0.35	8.04
Biomarkers of effects	Cells with Chromosomal Aberrations in culture at 48 h [≥1 Total cells with CA]	6.61	0.66	65.41
	Cells with Chromosomal Aberrations (unstable[Cu] e stable[Cs]) at 48 h [≥1 Total cells with CA]	0.08	0.00	0.81
	Cells (‰) with Micronuclei in culture at 48 h [≥2.00 cells (‰) with micronuclei]	3.67	0.65	20.62
	Cells with Chromosomal Aberrations in culture at 72 h [≥1 Total cells with CA]	0.93	0.19	4.58
	Cells with Chromosomal Aberrations ((Instable(Cu) e stable [Cs]) a 72 ore [≥1 Total cells with CA]	1.56	0.32	7.59
	Cells (‰) with Micronuclei in culture at 72 h [≥2.33 cells (‰) with micronuclei]	1.26	0.28	5.63
	Cells with Chromosomal Aberrations in culture at 72 h with Cytocala B [≥22.60 cells (‰) with micronuclei/total cells]	0.92	0.18	4.63
	Sister Chromatid Exchange (SCE) with TCR [≥377.84 sce/cells chromosomes]	0.69	0.11	4.09
	Sister Chromatid Exchange (SCE) with T [≥394.72 total sce]	1.86	0.20	17.09
	Sister Chromatid Exchange (SCE) with X [≥8.19 mean sce]	0.75	0.12	4.39
	Alterazioni del DNA Urinario (Flow Cytofluorimetry) [≥5.90]	0.40	0.08	1.90
	Mutations with Unscheduled DNA Synthesis (UDS) [≥1149.72 cpm/10^6^ cells]	2.19	0.45	10.49
	Cells with Thioguanine Resistance (TG^R^) [≥2.36 TG^R^ cells/10^6^ cells]	2.92	0.32	26.58
	Cells with Cloning Efficiency (CE) [≥13.30 %]	2.90	0.32	25.68
	Cells with Mutant Frequencies (MF) [≥25.48 MF/10^6^ cells ]	2.38	0.25	22.70
Biomarkers of genetical susceptibility	MSPI (CYPA1A variant Restriction enzyme Polymorphism) [Precence]	-	-	-
	ILEVAL (CYPA1A variant Isoleucine-Valine polymorphisms) [Presence]	-	-	-
	GSTM1 (Glutathione S-Transferase Mu 1 gene polymorphisms) [Presence]	-	-	-
	NAT2 (N-acetyltransferase 2 gene polymorphisms) [presence]	0.84	0.03	23.18

* All these estimates (ORs) were adjusted by pack years, night shift work, residence in Taranto Tamburi and charcoal-broiled food intake.

## Supplementary Materials

The following are available online at https://www.mdpi.com/1660-4601/17/7/2199/s1, Table S1: Frequency distribution of the main individual characteristics by study groups., Table S2: Vital status of the study groups at the end of study (2019), Table S3: Characteristics of Study Death Subject, Table S4: Environmental exposure concentrations, biomarkers measurements and polymorphysms frequencies by study groups, Table S5: Distributionn of adjusted estimates of risks (*ORs) to observe values of different biomarkers higher to the 66th centile among coke oven plant workers versus non coke oven plant workers, Table S6: Distributionn of adjusted estimates of risks (*ORs) to observe values of different biomarkers higher to the 66th centile by vital status (deaths for all the causes), Table S7: Distributions of adjusted estimates of risks (*ORs) to observe values of different biomarkers higher to the 66th centile by cancer death. Text S1: Job title description. Text S2: 3.4.1 Exposure measurements.
